# Activation of the habenula complex evokes autonomic physiological responses similar to those associated with emotional stress

**DOI:** 10.14814/phy2.12297

**Published:** 2015-02-12

**Authors:** Youichirou Ootsuka, Mazher Mohammed

**Affiliations:** Centre for Neuroscience, Department of Human Physiology, Flinders UniversityAdelaide, South Australia, Australia

**Keywords:** Body temperature, brown adipose tissue temperature, cutaneous blood flow, lateral habenula, thermoregulation

## Abstract

Neurons in the lateral habenula (LHb) discharge when an animal anticipates an aversive outcome or when an expected reward is not forthcoming, contributing to the behavioral response to aversive situations. So far, there is little information as to whether the LHb also contributes to autonomic physiological responses, including increases in body temperature (emotional hyperthermia) that are integrated with defensive behaviors. Vasoconstriction in cutaneous vascular bed and heat production in brown adipose tissue (BAT) both contribute to emotional hyperthermia. Our present study determines whether stimulation of the LHb elicits constriction of the tail artery and BAT thermogenesis in anesthetized Sprague–Dawley rats. Disinhibition of neurons in LHb with focal microinjections of bicuculline (1 nmol in 100 nl, bilaterally) acutely increased BAT temperature (+0.6 ± 0.1°C, *n* = 9 rats, *P* < 0.01) and reduced tail artery blood flow (by 88 ± 4%, *n* = 9 rats, *P* < 0.01). Falls in mesenteric blood flow, simultaneously recorded, were much less intense. The pattern of BAT thermogenesis and cutaneous vasoconstriction elicited by stimulating the habenula is similar to the pattern observed during stress-induced emotional hyperthermia, suggesting that the habenula may be important in this response.

## Introduction

The habenula complex, a highly conserved region of the diencephalon (Bianco and Wilson 2009), comprises two distinct nuclei, the lateral (LHb) and the medial habenula. Via the stria medullaris, the LHb receives diverse afferent information from a number of forebrain regions (Herkenham and Nauta 1977, 1979; Sutherland 1982). Via the fasciculus retroflexus, the LHb sends efferents to midbrain regions, including ventral tegmental area (VTA) and midbrain raphé monoaminergic systems important for autonomic control (Herkenham and Nauta 1977, 1979; Sutherland 1982). Neurons in LHb discharge when an animal anticipates an aversive outcome or when an expected reward is not forthcoming (Matsumoto and Hikosaka 2007, 2009; Hikosaka 2010).

Potentially threatening or aversive events also produce autonomic physiological responses (Korte et al. 2005; DiMicco et al. 2006). As well as changes in blood pressure, heart rate, and respiration, the autonomic response to an aversive or potentially aversive environment also includes thermoregulatory changes. The cutaneous vascular bed constricts and thermogenesis occurs in brown adipose tissue (BAT), resulting in a rise in body temperature referred to as emotional hyperthermia (Zaretsky et al. 2003; Kabir et al. 2010; Mohammed et al. 2014a). These thermoregulatory changes are mediated by the brainstem including an important control center in the raphé/parapyramidal region of the medulla oblongata (Zaretsky et al. 2003; Ootsuka and Blessing 2006). Physiological links connecting hypothalamic autonomic centers with the medulla oblongata are well established (DiMicco et al. 2006; Morrison and Nakamura 2011). However, at present, there is little information as to whether the LHb contributes to the autonomic components of the overall integrated behavioral response to aversive situations.

Both the behavioral and the autonomic components of the response to aversive stimuli are preceded by sudden increases in the proportion of theta (5–8 Hz) activity in the hippocampus (de Menezes et al. 2009; Buzsaki and Moser 2013). It is thus interesting to note the close link between the occurrence of hippocampal theta oscillations and activity of LHb neurons (Aizawa et al. 2013). Thus, the anatomical and functional evidences suggest the possibility that the LHb may contribute to the link between forebrain emotional systems and brainstem regions mediating autonomic physiological changes. To test this hypothesis, we determined whether activation of the habenula complex, focusing on the LHb, elicits autonomic responses similar to those observed during emotional hyperthermia, focusing on vasoconstriction of the thermoregulatory tail vasculature and BAT thermogenesis in rat (de Menezes et al. 2009; Mohammed et al. 2014a,b).

## Material and Methods

### Surgical procedures

All experiments were performed in male Sprague–Dawley rats (300–400 g, *n* = 15) with procedures approved by the Animal Welfare Committee of Flinders University. There were two groups; blood flow-recording group and BAT nerve-recording group.

In the blood flow-recording group (*n* = 9), preparatory surgery was performed to implant tail and mesenteric artery Doppler flow probes under isoflurane inhalation (2%) (Veterinary Companies of Australia, Australia) (de Menezes et al. 2009; Mohammed et al. 2014a). At the end of the surgery, antibiotic (Baytril, 15 mg/kg s.c. Bayer Australia, Pymble, NSW, Australia) and analgesic (carprofen, 5 mg/kg s.c., Carprieve, Norbrook Laboratories Australia PTY Ltd, Tullamarine, Vic., Australia) were administered. All rats were then caged individually and had at least 1-week recovery before experiments.

On the day of experiment, rats were anesthetized with isoflurane (2% in oxygen), and an endotracheal tube was inserted via a tracheotomy. The right femoral vein and artery were cannulated for injection of drug and measurement of arterial pressure, respectively. The level of anesthesia was maintained at a depth sufficient to abolish paw withdrawal reflexes. Rats were then mounted prone on stereotaxic setup (David Kopf Instruments, Tujunga, CA). Burr-hole craniotomy was performed for electrical stimulation or bicuculline injection into left and right LHb (3.6 mm caudal from Bregma, 0.7 mm lateral from midline, and 4.6 mm deep from cortex surface).

In the blood flow-recording group, during the course of experiments, rats were paralyzed with d-tubocurarine (initially 0.3 mg i.v. [1–1.6 mg/kg], thereafter, 0.3 mg i.v. every 1–1.5 h) (Sigma-Aldrich Co, Castle Hill, NSW, Australia) and ventilated artificially with 100% O_2_ (60–65 cycle/min, 2–3 ml/cycle). The animal was allowed to recover from paralysis between doses, so that adequate anesthesia could be confirmed before paralysis was reestablished.

In the BAT nerve-recording group (*n* = 6), no preparatory surgery was performed. On the day of experiment, rats were initially anesthetized with isoflurane (2% in oxygen), and then replaced with a cocktail of *α*-chloralose (40–80 mg/kg, i.v.) and urethane (400–800 mg/kg) after tracheotomy and cannulation of the femoral artery and vein. The rats were paralyzed with d-tubocurarine and ventilated artificially with 100% O_2_. Burr-hole craniotomy was performed for electrical stimulation into LHb. Interscapular BAT sympathetic nerve was isolated as described previously (Ootsuka and McAllen 2006). Nerve activity was recorded by a pair of silver electrode and amplified (x20,000, NL104, Digitimer Ltd., Welwyn Garden City, U.K.) and filtered (band-pass 1–1000 Hz, NL125, Digitimer). At the end of experiments, a ganglionic blockade, chlorisondamine chloride (10 mg/kg i.v.) was administered to confirm a loss of BAT sympathetic nerve activity and thus to ensure that nerve recording was from postganglionic sympathetic axons.

Tail and mesenteric artery Doppler blood flow signals were monitored using System 6 Model (Triton Technology, Mansfield, MA). End expiratory CO_2_ (ExpCO_2_) was maintained at 4–5% at resting condition (Normocap; Datex, Helsinki, Finland). The BAT, rectal (body), and abdominal skin temperatures were measured with thermocouples (TC-2000; Sable Systems, Las Vegas, NV). Arterial pressure was measured using transducer (P23, Gould Inc, Oxnard, CA) and bridge amplifier (7P112C, Grass Instrument Co, Quincy, MA). Pupil dilatation was assessed qualitatively by visual examination.

### Experimental procedures

In the blood flow-recording group (*n* = 9), firstly, the LHb was electrically stimulated with a glass-insulated tungsten electrode (50–100 *μ*m of tip exposed) (0.5–1 mA, 1 ms pulses duration, 20 ms separation for 10 sec). In eight of the nine rats, control electrical stimulations were made 2 mm ventral, 2 mm dorsal, and 2 mm lateral to the LHb. Then, a glass micropipette filled with either ringer or bicuculline ((-)-bicuculline methiodide, Tocris Bioscience, Bristol, U.K.) was inserted in the LHb site. Ringer vehicle (100 nl, *n* = 9) was firstly injected into the LHb, and the response recorded. After at least 10 min, 100 nl of bicuculline (1 nmol, *n* = 9) was injected into the LHb bilaterally.

After approximately 1 h later, when tail flow returned to preinjection level, seven of the nine rats were paralyzed and ventilated artificially to exclude possible secondary effects of muscle contraction. Then, another bilateral injection of bicuculline was made into the LHb (*n* = 7). In a subgroup of the seven paralyzed rats, bicuculline was also injected into either the third ventricle (*n* = 4) or lateral thalamus at 2 mm lateral (*n* = 6) to the LHb. At least 10-min observation time was put after each injection even if no response was observed.

In the BAT nerve-recording group (*n* = 6), only electrical stimulation was made in the LHb. BAT sympathetic nerve response to the stimulation was assessed using peri-stimulus time histogram.

### Histology

At the end of experiment, rat was deeply anesthetized with pentobarbitone sodium (over 80 mg/kg i.v.) and perfused transcardially with saline followed by formaldehyde-fixatives (4%) contained picric acid (15%). The brain was removed for histological confirmation of injection site by visualization of *β*-galactosidase reaction product or horseradish peroxidase (HRP) reaction products (*β*-galactosidase or HRP was added to the drug injectate). For the HRP reaction, the brain sections were incubated in 0.05% diaminobenzidine (DAB) solution (in 0.1 M Tris buffer saline) for 10 min and then hydrogen peroxide was added to the incubation solution (0.37 *μ*l of 30% H_2_O_2_ per 1 ml of the DAB solution). For the *β*-galactosidase reaction, the brain sections were incubated in 0.1% X-Gal solution (0.1% X-Gal, 10 mM NaH_2_PO_4_, 1 mM MgCl_2_, 150 mM NaCl, 3.3 mM K_4_Fe(CN)_6_3H_2_O, 3.3 mM K_3_Fe(CN)_6_) for 2–3 h.

### Data analysis

All data were sampled and digitized by PowerLab at 1 kHz (ADInstruments Inc., Bella Vista, NSW, Australia). Heart rate was computed from arterial pressure or blood flow. Respiratory rate was computed from pulsatile instantaneous changes in ExpCO_2_. Our direct measurement of instantaneous change in blood flow with pulse Doppler system enables us to calculate amplitude of pulsate in blood flow. Assessing flow pulse amplitude rather than calculating conductance with mean blood flow and mean arterial pressure is a simpler and more direct approach to evaluate neutrally mediated vasoconstriction. Data were analyzed with Igor Pro (WaveMetrics, Portland, OR), and with StatView (SAS Institute, Cary, NC). Group data were shown as means ± SE. The statistical significance of mean differences between pre- and poststimulation/injection values and between tail and mesenteric flow variables were calculated and compared using student's *t*-test. Significance level was set at the *P* < 0.05.

## Results

Electrical stimulation in the LHb (*n* = 9) decreased tail blood flow by 78 ± 4%, and pulse amplitude by 27 ± 6% (*P* < 0.01), indicating strong vasoconstriction. The stimulation also decreased mesenteric artery flow by 68 ± 5%, and pulse amplitude by 29 ± 3% (*P* < 0.01). The mesenteric flow and amplitude reductions were significantly less than those from the tail (*P* < 0.01). The LHb stimulation increased arterial pressure (by 24 ± 3 mmHg, *P* < 0.01), heart rate (by 21 ± 5 bpm, *P* < 0.01), and respiratory rate (by 19 ± 8 cpm, *P* < 0.01). There were no significant changes in brown adipose tissue (BAT) and body temperature and ExpCO_2_. The electrical stimulation also elicited pupil dilatation, as observed by direct inspection. No detectable bodily movement was observed during the stimulation.

Electrical stimulation in 2 mm ventral to the LHb (*n* = 8) decreased tail blood flow by 60 ± 7%, and pulse amplitude by 42 ± 8% (*P* < 0.01). The stimulation also decreased mesenteric artery flow by 34 ± 5%, but increased its pulse amplitude by 11 ± 3% (*P* < 0.01). The ventral stimulation increased arterial pressure by 30 ± 6 mmHg (*P* < 0.01). Heart rate and respiratory rate did not change.

Electrical stimulation 2 mm dorsal to the LHb (in the hippocampal formation, *n* = 8) caused a slight fall in arterial pressure (by 6 ± 2 mmHg, *P* < 0.01) and in tail blood flow (by 24 ± 6%, *P* < 0.05) without a change in pulse amplitude, indicating no significant vasoconstriction. The hippocampal stimulation caused a slight fall in heat rate (by 3 ± 1 bpm, *P* < 0.05). Electrical stimulation in 2 mm lateral to the LHb caused a slight fall in arterial pressure (by 4 ± 2 mmHg, *P* < 0.05). The other parameters did not change.

**Figure 1:**
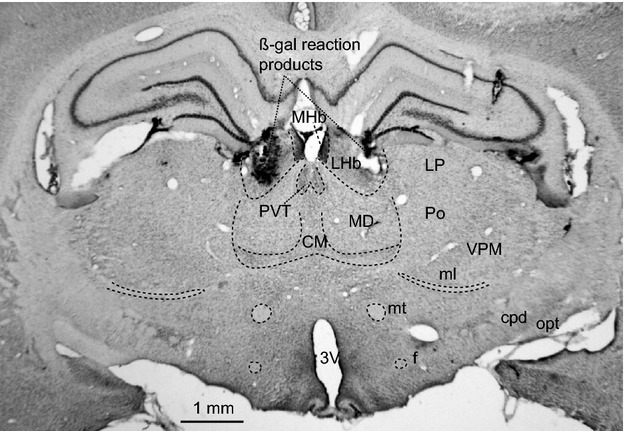
Histological demonstration of bicuculline injection sites into LHb marked by ß-gal reaction products. CM, central medial thalamic nucleus; cpd, cerebral peduncle; f, fornix; LHb, lateral habenula; MD, mediodorsal thalamic nucleus; MHb, medial habenula; ml, medial lemniscus; mt mammillothalamic tract; LP, lateral posterior thalamic nucleus; opt, optic tract; Po, posterior thalamic nucleus; PVT, paraventricular thalamic nucleus; VPM, ventral posteromedial thalamic nucleus; 3V third ventricle.

**Figure 2:**
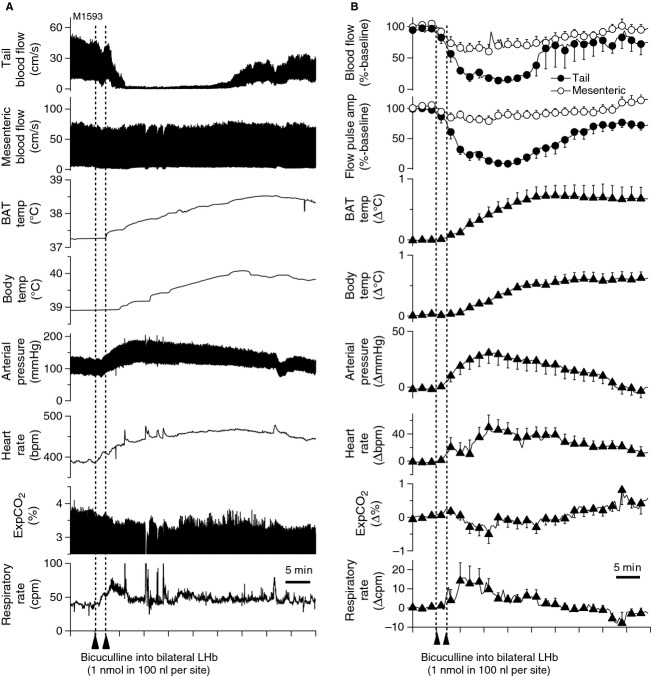
Effect of bicuculline injection into LHb on tail and mesenteric blood flow, and their flow pulse amplitude, brown adipose tissue and body temperature (BAT temp, Body temp), arterial pressure, heart rate, end expiratory CO_2_ (ExpCO_2_), and respiratory rate under both nonparalysis. (A) Chart record from one anesthetized rat (B) Group data. Relative percentage difference from preinjection baseline level was shown in tail and mesenteric flow. Actual difference (Δ) from preinjection baseline level was shown in all other parameters.

After bicuculline injection, into the LHb (Fig.1) in nonparalyzed condition (*n* = 9), tail blood flow started decreasing within 1 min and reached minimum values within 10 min (Fig.2). The reduction of tail flow and its pulse amplitude were significantly larger than those of mesenteric flow (Table1). The bicuculline injection also elicited a significant increase in BAT and body temperatures and arterial pressure. Pupil dilatation was observed. Respiratory rate did not change. ExpCO_2_ increased in paralyzed condition (from 3.8 ± 0.2 to 4.0 ± 0.2% *P* < 0.05, *n* = 7). In subgroups of the seven rats in paralyzed condition, similar bicuculline injections into the third ventricle (*n* = 4) or into lateral thalamus (2 mm lateral to the LHb, *n* = 6) did not cause significant changes in any recorded parameter (Table1).

**Table 1:** The effect of ringer or bicuculline injection into LHb and adjacent areas on physiological parameters.

	Ringer	Bicuculline			
	LHb (9)	LHb (9) nonparalysis	LHb (7) paralysis	Lateral (6)	3rd ventricle (4)
Tail flow (%)					
Before	100	100	100	100	100
After	103 ± 6	17 ± 4^*^††	12 ± 4^*^^*^††	98 ± 9	77 ± 16
Pulse					
Before	100	100	100	100	100
After	99 ± 1	11 ± 3^*^††	8 ± 3^*^^*^††	95 ± 7	74 ± 18
Mes flow (%)					
Before	100	100	100	100	100
After	99 ± 1	71 ± 8^*^	83 ± 9	106 ± 5	101 ± 2
Pulse					
Before	100	100	100	100	100
After	102 ± 3	85 ± 7	73 ± 6^*^^*^	92 ± 2	117 ± 5
BAT (°C)					
Before	37.4 ± 0.2	37.3 ± 0.2	37.6 ± 0.2	37.7 ± 0.2	38.1 ± 0.2
After	37.3 ± 0.3	37.9 ± 0.2^*^^*^	38.1 ± 0.2^*^^*^	37.6 ± 0.2	38.2 ± 0.2
Body (°C)					
Before	38.9 ± 0.2	38.9 ± 0.7	39.6 ± 0.6	39.4 ± 0.2	40.2 ± 0.3
After	39.0 ± 0.2	39.4 ± 0.2^*^^*^	39.9 ± 0.2^*^^*^	39.4 ± 0.2	40.4 ± 4
AP (mmHg)					
Before	90 ± 3	88 ± 3	84 ± 5	84 ± 6	74 ± 7
After	90 ± 3	113 ± 7^*^	119 ± 8^*^^*^	88 ± 6	74 ± 7
HR (bpm)					
Before	383 ± 7	373 ± 6	385 ± 7	380 ± 12	408 ± 10
After	382 ± 7	415 ± 15^*^	403 ± 21	375 ± 6	408 ± 11

Value in brackets is the number of rats. pulse, mean amplitude of pulsatory blood flow; BAT, brown adipose tissue temperature; Body, body temperature; AP, arterial pressure; HR, heart rate. ^*^P < 0.05, ^*^^*^P < 0.01, significant different from mean values during 5 min before injection (before). ††*P* < 0.01 significant different from the corresponding value from mesenteric artery (Mes).

**Figure 3:**
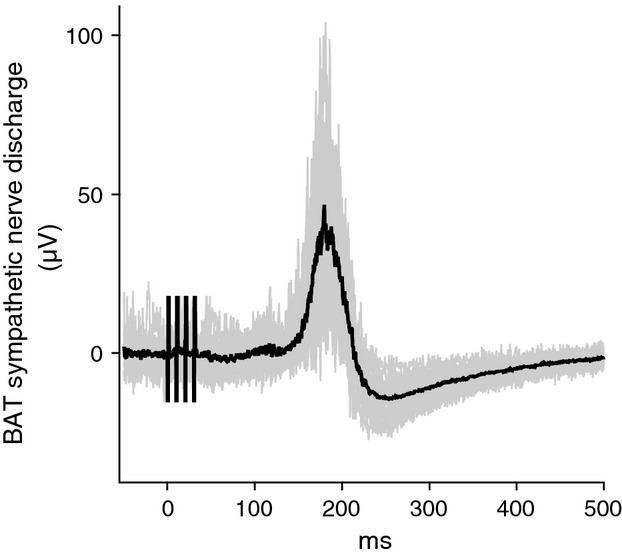
Peri-stimulus averaged potentials of BAT sympathetic nerve evoked by electrical stimulation of LHb at time 0. Gray lines show 16 individual sweeps and black line shows an average of the 16 sweeps.

In the BAT nerve-recording group, electrical stimulation of the LHb (4 pulses, 10-ms interval, 1-ms duration, every 10 sec) evoked a vigorous discharge in the BAT sympathetic nerve, with an onset latency of 159 ± 8 ms and a peak latency of 194 ± 9 ms (*n* = 6) (Fig.3).

## Discussion

Our study reports for the first time that stimulation of the habenula complex produces integrated autonomic responses in rats, including activation of sympathetically innervated thermoregulatory end organs. Both electrical stimulation and bicuculline-mediated excitation of neuronal perikarya in this nucleus elicited strong vasoconstriction in the thermoregulatory cutaneous vascular bed. Vasoconstriction in the mesenteric bed, simultaneously measured, was much less intense. The bicuculline-mediated excitation of neurons in the habenula complex also elicited BAT thermogenesis. Recordings from BAT sympathetic nerves confirmed that the BAT thermogenesis was sympathetically mediated. CO_2_ production increased in the paralyzed artificially ventilated animal, confirming an overall increase in metabolic rate.

In conscious rats, the observed thermoregulatory response pattern normally occurs in association with emotional stress (Mohammed et al. 2014a). When animals are exposed to salient or emotionally significant situations, strong vasoconstriction is elicited in the cutaneous vascular bed, with less response in the mesenteric bed (Yu and Blessing 1999; de Menezes et al. 2009). BAT thermogenesis, together with the cutaneous vasoconstriction contributes to the emotional hyperthermia (Mohammed et al. 2014a). Emotional stress also causes tachycardia and pressor responses (Carrive 2002; Zaretsky et al. 2003; Kabir et al. 2010), as also noted in our study. The habenula-elicited autonomic physiological responses we observed are also similar to those occurring during the ‘defense reaction’ (Hess 1981). A previous study, also in anaesthetized rats, reported that unilateral injection of L-glutamate into the habenula complex increases blood pressure and heart rate (Gao and Wang 1988), and similar responses also were observed in our study. Our electrical stimulation and drug injection targeted the LHb. Nevertheless, we acknowledge that the method used in the present study cannot fully differentiate between the medial and lateral subnuclei of the habenula complex.

There are few or no intrinsic GABAergic neurons in the LHb (Belin et al. 1982; Mugnaini and Oertel 1985; Smith et al. 1987). Thus, the bicuculline-elicited autonomic responses are presumably mediated by blockade of GABAergic sources to the LHb from other brain areas, possibly the entopeduncular nucleus or the lateral preoptic area (Araki et al. 1984). Inhibition of these areas evokes BAT thermogenesis (de Luca et al. 1989) and cutaneous vasoconstriction (Tanaka et al. 2009).

The medullary raphé/parapyramidal region, at the rostrocaudal level of the caudal third of the facial nucleus, is a lower brainstem thermoregulatory control center, relaying excitatory input to spinal sympathetic neurons regulating BAT and the tail cutaneous vascular bed (Smith et al. 1998; Oldfield et al. 2002; Samuels et al. 2002; Cao and Morrison 2003; Nakamura et al. 2004; Ootsuka et al. 2004). Inhibition of neuronal function in this raphé/parapyramidal region using local microinjections of muscimol entirely prevents excitation of BAT sympathetic nerves elicited from stimulation of the LHb (Y. Ootsuka and M. Mohammed, unpubl. obs.). It is thus likely that output to the spinal sympathetic neurons from the LHb involves a relay via raphé/parapyramidal neurons.

The major output from the LHb is the fasciculus retroflexus (Quina et al. 2014). Via this pathway, axons from the LHb innervate midbrain areas including the rostromedial mesopontine tegmental nucleus and the VTA, dorsal, median, and pontine raphé nuclei, as well as thalamic, hypothalamic, and basal forebrain structures (Herkenham and Nauta 1979; Araki et al. 1988; Poller et al. 2011; Bernard and Veh 2012; Quina et al. 2014). The medullary raphé/parapyramidal region receives direct efferent connections from subsets of these areas (Hermann et al. 1997). Activation of hypothalamic centers and midbrain evokes BAT thermogenesis (Amir 1990; Dib et al. 1994; Cerri and Morrison 2005) and cutaneous vasoconstriction (Zhang et al. 1997; Nalivaiko and Blessing 2001; Tanaka and McAllen 2008). However, the detailed pathways mediating the thermoregulatory responses to LHb stimulation remain to be established. Cutaneous vasoconstriction and BAT thermogenesis also occur in response to thermoregulatory stress such as exposing to cold or to pyrogenic agents (Ootsuka and McAllen 2006; Ootsuka et al. 2008). The preoptic area and the dorsomedial hypothalamic area are involved in these thermoregulatory responses (Morrison and Nakamura 2011). The LHb might be involved in the thermoregulatory response mediated by those areas.

There are no or very few direct connections to the medullary raphé/parapyramidal thermoregulatory control center from the LHb (Araki et al. 1988; Quina et al. 2014). In this study, the mean onset latency of the excitatory potential evoked in BAT sympathetic nerve discharges by the LHb stimulation was about 160 ms. This is substantially longer than 140 ms, the corresponding latency observed with medullary raphé/parapyramidal stimulation (Morrison 1999). The latency difference strongly suggests polysynaptic descending pathways from the LHb to the medullary raphé/parapyramidal region, in agreement with the neuroanatomical studies cited above.
